# Rapid Identification of *Candida* Species in Candidemia Directly from Blood Samples Using Imperfect Match Probes

**DOI:** 10.1038/s41598-020-62276-5

**Published:** 2020-04-02

**Authors:** Yoshitsugu Higashi, Hideki Niimi, Ippei Sakamaki, Yoshihiro Yamamoto, Isao Kitajima

**Affiliations:** 1 Department of Clinical Infectious Diseases, Faculty of Medicine, Academic Assembly, University of Toyama, Toyama, 930-0194 Japan; 2 Department of Clinical Laboratory and Molecular Pathology, Faculty of Medicine, Academic Assembly, University of Toyama, Toyama, 930-0194 Japan

**Keywords:** Fungal infection, Laboratory techniques and procedures

## Abstract

Candidemia is associated with a high mortality rate, and initial adequate antifungal therapy results in a significant decrease in the crude mortality. We herein report a rapid method that can identify eight *Candida* species in candidemia using imperfect match quenching probes (IM Q-probes) within three and a half hours of whole blood sample collection. Furthermore, employing the D value, which reflects the difference between the Tm signature from a clinical isolate and that registered in the database, it is possible to quickly identify samples suitable for IM Q-probe identification. We first evaluated the method using 34 *Candida* colonies collected from different patients, and 100% (34/34) of the identification results matched the preidentified *Candida* species. We then performed blind tests using eight whole blood samples artificially mixed with eight different *Candida* species respectively, and all identification results correctly matched the preidentified *Candida* species. Finally, using 16 whole blood samples collected from candidemia patients, we compared the IM Q-probe method with the culture/sequencing method. Of a total of 16 patient samples, 100% (16/16) matched the culture and sequencing results. The IM Q-probe method is expected to contribute not only to the life expectancy of candidemia patients but also to antifungal stewardship.

## Introduction

*Candida* species are the most frequent organisms involved in invasive fungal infections and major causes of not only local mucous membrane infections but also widespread propagation with multisystem organ failure^1^. Recently, due to advances in medicine, such as dialysis, major surgery and immunosuppressants, the rates of invasive candidiasis have been increasing. Indeed, in the United States, *Candida* species are the fourth-most common pathogen of nosocomial bloodstream infection^2,3^. Candidemia has an associated mortality that is as high as 25%, and initial adequate antifungal therapy results in a significant decrease in the crude mortality^4–6^. However, blood cultures usually take two to five days to finalize, which leads to a significant delay in the implementation of culture-driven therapy^1,7,8^. In addition, although most episodes of candidemia are caused by *Candida albicans*, opportunistic infections due to non-*C. albicans* species have been reported with increasing frequency^9^.

Pfaller *et al*. reported that the trends in descending order of frequency of *Candida* species causing candidemia were *C. albicans* (42.1%), *C. glabrata* (26.7%), *C. parapsilosis* (15.9%), *C. tropicalis* (8.7%), *C. krusei* (3.4%), *C. lusitaniae* (1.1%), *C. dubliniensis* (0.9%) and *C. guilliermondii* (0.4%)^10^. Because definitive therapy by selecting an appropriate anti-*Candida* agent should be based on the species of *Candida*, the rapid identification of *Candida* species is critical for the prompt initiation of appropriate therapy^1^.

Niimi *et al*. reported the novel “melting temperature mapping method” for rapidly identifying the dominant bacteria in a clinical sample (whole blood sample, etc.) from a sterile site^11^, which is our previous teamwork. This study suggested that more than 100 bacterial species can be identified by using the melting temperatures (Tm) of 7 primer sets, and these identification results can be obtained within three hours of whole blood collection. This method is useful for identifying bacteria. However, regarding the identification of *Candida* species, it is more difficult to design universal primers for fungi than for bacteria since *Candida* species are eukaryotes, like human cells. In addition, to identify many *Candida* species at the species level, specific primers must be generally prepared for each *Candida* species. As a result, the system becomes more complicated.

To address these problems, we herein report the development of a rapid and easy method for identifying *Candida* species in candidemia using a real-time polymerase chain reaction (PCR)-based system.

## Results

### Workflow of the rapid identification method for eight *Candida* species

**Figure 1:**
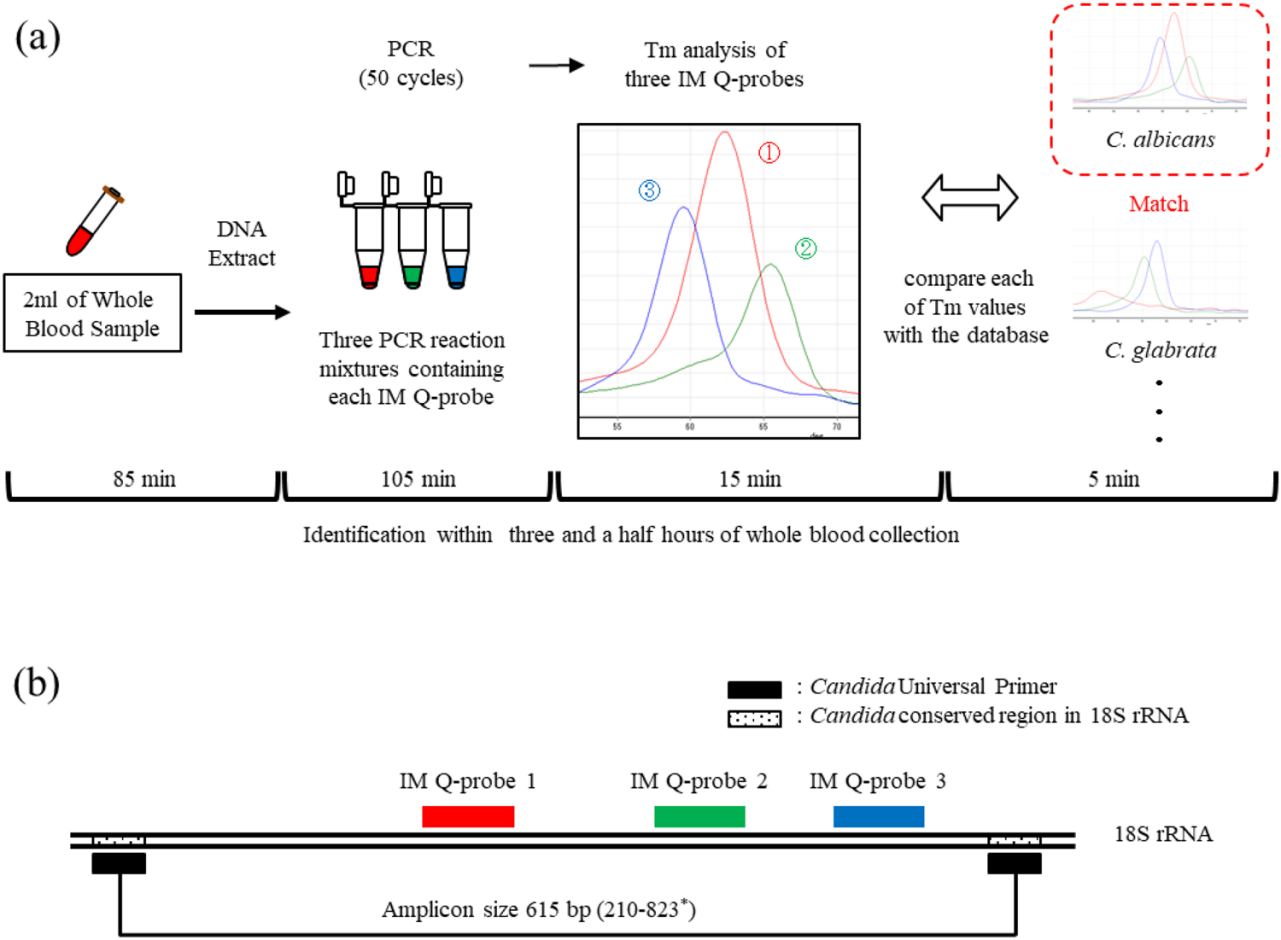
Workflow of the rapid identification method for eight *Candida* species within three and a half hours of sample collection. (**a**) Using a *Candida* universal primer and three IM Q-probes, this method rapidly identifies eight *Candida* species by finding the nearest matches of the Tm values in the database. (**b**) We designed three IM Q-probes that can bind the position with some probe-target mismatches in the *Candida* universal PCR amplicon. * *Candida albicans* 18 S ribosomal RNA gene (Accession No. AF114470).

The workflow of the rapid identification method for *Candida* species developed in our laboratory is shown in Fig. 1a. Employing *Candida* universal PCR (using one *Candida* universal primer) and a melting temperature (Tm) value analysis of three probes, eight *Candida* species are able to be identified within three and a half hours of whole blood collection.

**Figure 2:**
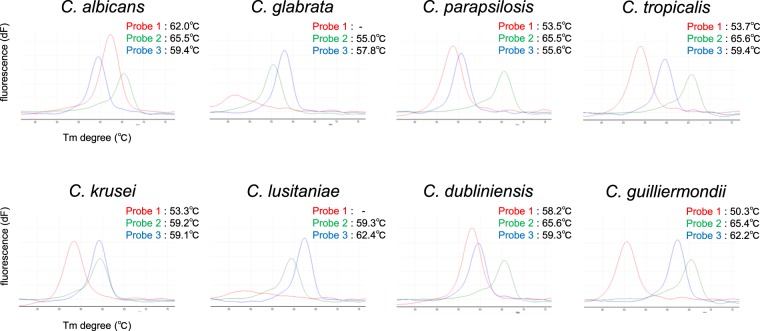
The variations in the three melting peak patterns among the eight *Candida* species. Red melting peak: IM Q-probe 1, green melting peak: IM Q-probe 2, blue melting peak: IM Q-probe 3.

The eight *Candida* species causing candidemia were selected based on the trends in descending order of frequency, as described above^10^. This method consists of four major steps. First, fungal DNA is directly extracted from a clinical sample (e.g. 2 mL of whole blood sample) as a template for PCR. Step two involves PCR using a *Candida* universal primer and three imperfect match quenching probes (IM Q-probes). The *Candida* universal primer can detect all species of *Candida*. The three PCR reaction mixtures (three PCR tubes) contain the same *Candida* universal primer set and each IM Q-probe. We designed three IM Q-probes (IM Q-probe 1, IM Q-probe 2, IM Q-probe 3) that are able to bind to the positions with some probe-target base sequence mismatches in the same *Candida* universal PCR amplicon (Fig. 1b, Table 1). Allowing for some probe-target base sequence mismatches, there is substantial flexibility in these IM Q-probe designs. To obtain stable Tm values (and therefore stable identification results), we carefully selected each probe position whose target base sequence is the same among the different strains of the same *Candida* species reported in the DNA Data Bank of Japan (Table S1). PCR is then performed, and one PCR amplicon is obtained. In step three, the three Tm values are acquired by analyzing each IM Q-probe. Each of the Tm values varies depending on *Candida* species. The Tm value analysis showed various melting peaks (i.e. Tm values) among the eight *Candida* species with each IM Q-probe (Fig. S1). Consequently, the combination of the three Tm values was *Candida* species-specific (Fig. 2). In *C. glabrata* and *C. lusitaniae*, using the IM Q-probe 1, Tm values could not be obtained because of the large number of probe–target mismatches. Step four involves the identification of *Candida* species by comparing each of three Tm values with the database. We constructed a preliminary database of the eight *Candida* species showing the mean values of triplicate Tm measurements (Table S2). *Candida* isolates are able to be rapidly identified by comparing the Tm values to the values in the database.

**Table 1:** Sequence homology between the IM Q-probes and the target regions in the eight *Candida* species.

*Candida* species		IM Q-probe 1 (5′→3′)	Mismatch	IM Q-probe 2 (5′→3′)	Mismatch	IM Q-probe 3 (5′→3′)	Mismatch
		CTTTCCTTCTGGGTAGCCATTT	(bp)	TGGAATAATAGAATAGGACGTTATGGTTC	(bp)	GCATCAGTAATCAGTTGTCAGAGGAGAAATTC	(bp)
*Candidaalbicans*	(AF114470)	CTTTCCTTCTGGGTAGCCATTT	0	TGGAATAATAGAATAGGACGTTATGGTTC	0	GTATCAGTATTCAGTTGTCAGAGGTGAAATTC	3
*Candida glabrata*	(AY083231)	CTTTCCTTCTGGCTAACCCCAA	6	TGGAATAATGGAATAGGACGTT-TGGTTC	2	GCATCAGTATTCAATTGTCAGAGGTGAAATTC	3
*Candida parapsilosis*	(AB030915)	CTTTCCTTCTGGCTAGCCTTTT	2	TGGAATAATAGAATAGGACGTTATGGTTC	0	GTATCAGTATTCAGTAGTCAGAGGTGAAATTC	4
*Candida tropicalis*	(KT449837)	CTTTCCTTCTGGCTAGCCTTTT	2	TGGAATAATAGAATAGGACGTTATGGTTC	0	GTATCAGTATTCAGTTGTCAGAGGTGAAATTC	3
*Candida krusei*	(AB053239)	CTTTCCTTCTGGCTAGCCCTCG	4	TGGAATAATAGAATAGGACGC-ATGGTTC	2	GCATCAGTATTCAGTCGTCAGAGGTGAAATTC	3
*Candida lusitaniae*	(KU147480)	CTTTCCTCCTCCTCTTAGCAAT	12	TGGAATAATAGAATAGGACGC-ATGGTTC	2	GCATCAGTATTCAGTTGTCAGAGGTGAAATTC	2
*Candida dubliniensis*	(AY497766)	CTTTCCTTCTGGCTAGCCATTT	1	TGGAATAATAGAATAGGACGTTATGGTTC	0	GTATCAGTATTCAGTTGTCAGAGGTGAAATTC	3
*Candida guilliermondii*	(AY497770)	CTTTCCTTCTGGCTAACCATTC	3	TGGAATAATAGAATAGGACGTTATGGTTC	0	GCATCAGTATTCAGTTGTCAGAGGTGAAATTC	2

The base sequence differences between the IM Q-probes and the target regions are underlined.

**Figure 3:**
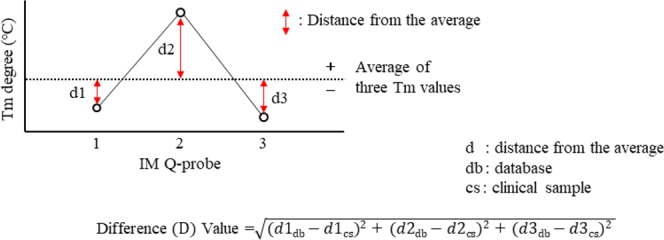
Difference (D) value. In order to identify a *Candida* isolate, we calculate the difference (D) value using the indicated formula. *Candida* species are identified by comparing the three distances from the average of the *Candida* isolate to those in the database.

In order to identify a *Candida* isolate, we calculate the difference (D) value using the formula shown in Fig. 3. The D value reflects the difference between the Tm signature from a clinical isolate and that registered in the database. *Candida* species are identified by comparing the three distances from the average of a *Candida* isolate to those in the database. Tm values above the average receive a “+” designation, while those below the average receive a “−” designation. The known *Candida* species in the database with the smallest D value is then identified as a correct match.

### The assessment of the accuracy of the IM Q-probe method

In order to assess the accuracy of the IM Q-probe method, we first validated the measurement error. Using the Rotor-Gene Q instrument, whose tube-to-tube variation is within ± 0.1 °C, we tried to validate the measurement errors among 10 samples using the same *C. albicans* DNA template in the same trial (Table S3). The results showed that the maximum measurement error of the IM Q-probes was ± 0.25 °C. On receiving this result, we then established preliminary interpretative criteria to assess the suitability of the IM Q-probe identification. The interpretative criteria for identification based on the D value are shown in Table 2. Considering the maximum measurement error of the IM Q-probes, identification results with a D value of ≤ 0.41 are considered the *Candida* isolate. To determine the D threshold (= 0.41), we calculated it employing three Tm degrees (+0.25, −0.25, +0.25) and the formula (Fig. 3). If the IM Q-probe method shows identification results with D values of >0.41, the identification result with the lowest D value is highly likely to be the *Candida* isolate due to the high specificity of the IM Q-probe identification (Table S4). *C. lusitaniae* and *C. glabrata* are narrowed down as pathogen, but cannot be distinguished using the D value only. In this case, it is easy to distinguish them by checking the difference in their Tm values already obtained, which are apparently different (Table S4). In addition, using the current protocols, the limit of identification of the IM Q-probe method is 1.2 CFU of *C. albicans* per PCR tube (Table S5).

**Table 2:** Interpretative criteria.

D	Suitability for identification	Identification	Interpretation
0.0 ≤ D ≤ 0.41	High	The identification result within this range is the *Candida* isolate*	completely matched the *Candida* species registered in the database to the base sequence level
0.41 < D	Medium to Low	The identification result with the lowest D is highly likely to be the *Candida* isolate	Mutant strain, polymicrobial infection, or not registered in the database

D, difference value.

*C. lusitaniae and *C. glabrata* are narrowed down as pathogen, but cannot be distinguished using the D value only. In this case, it is easy to distinguish them by checking the difference in their Tm values already obtained, which are apparently different (Table S4).

We subsequently evaluated the accuracy of the IM Q-probe method using 34 *Candida* colonies (8 *Candida* species) obtained from various type of samples (blood, sputum and urine) collected from different patients (Table 3). As a result, 100% (34/34) of the IM Q-probe results, whose D values were all ≤ 0.41, correctly matched the pre-identified *Candida* species by the sequencing method.

**Table 3:** Individual results of identification starting from *Candida* colonies.

Colonies of *Candida* species*	Tm degree (°C)			D Value	IM Q-probe Identification results
	IM Q-probe 1	IM Q-probe 2	IM Q-probe 3		
*C. albicans*	62.2	65.5	59.3	0.22	*C. albicans*
*C. albicans*	62.0	65.4	59.1	0.22	*C. albicans*
*C. albicans*	62.3	65.5	59.3	0.29	*C. albicans*
*C. albicans*	62.2	65.5	59.5	0.14	*C. albicans*
*C. albicans*	62.2	65.5	59.5	0.14	*C. albicans*
*C. albicans*	62.3	65.5	59.3	0.29	*C. albicans*
*C. albicans*	62.2	65.5	59.2	0.28	*C. albicans*
*C. albicans*	62.3	65.5	59.5	0.22	*C. albicans*
*C. albicans*	62.4	65.4	59.7	0.37	*C. albicans*
*C. glabrata*	—	54.7	57.7	0.14	*C. glabrata*
*C. glabrata*	—	55.0	57.8	0	*C. glabrata*
*C. glabrata*	—	54.9	57.7	0	*C. glabrata*
*C. glabrata*	—	54.9	57.7	0	*C. glabrata*
*C. glabrata*	—	55.0	57.8	0	*C. glabrata*
*C. parapsilosis*	53.5	65.2	55.5	0.22	*C. parapsilosis*
*C. parapsilosis*	53.2	65.3	55.3	0.08	*C. parapsilosis*
*C. parapsilosis*	53.4	65.4	55.4	0.08	*C. parapsilosis*
*C. parapsilosis*	53.4	65.4	55.6	0	*C. parapsilosis*
*C. parapsilosis*	53.4	65.4	55.7	0.16	*C. parapsilosis*
*C. tropicalis*	53.4	65.5	59.3	0.16	*C. tropicalis*
*C. tropicalis*	53.4	65.5	59.4	0.22	*C. tropicalis*
*C. tropicalis*	53.5	65.5	59.5	0.22	*C. tropicalis*
*C. krusei*	53.0	59.2	59.0	0.22	*C. krusei*
*C. krusei*	53.0	59.2	59.3	0.36	*C. krusei*
*C. krusei*	53.2	59.0	59.0	0.08	*C. krusei*
*C. lusitaniae*	—	59.2	62.3	0	*C. lusitaniae*
*C. lusitaniae*	—	59.2	62.2	0	*C. lusitaniae*
*C. lusitaniae*	—	59.3	62.3	0.07	*C. lusitaniae*
*C. dubliniensis*	58.0	65.5	59.2	0.08	*C. dubliniensis*
*C. dubliniensis*	58.2	65.5	59.2	0.08	*C. dubliniensis*
*C. dubliniensis*	58.0	65.5	59.5	0.29	*C. dubliniensis*
*C. guilliermondii*	50.2	65.2	62.1	0.08	*C. guilliermondii*
*C. guilliermondii*	50.2	65.4	62.1	0.08	*C. guilliermondii*
*C. guilliermondii*	50.3	65.5	62.3	0.08	*C. guilliermondii*

*These *Candida* species were identified using the sequencing method.

In order to assess the IM Q-probe method, we then performed blind tests using the whole blood samples artificially mixed with *Candida* species (200 CFU of *Candida* species per 2 mL of healthy whole blood). After concealing the name of *Candida* species, we tried to identify the *Candida* species in the whole blood. In all eight trials with eight different *Candida* species respectively, the IM Q-probe results, whose D values were all ≤ 0.41, correctly matched the pre-identified *Candida* species by the sequencing method (Table 4).

**Table 4:** The results of identification starting from whole blood samples artificially mixed with *Candida* species.

*Candida* species* in blood sample	Tm degree (°C)			D Value	IM Q-probe Identification results
	IM Q-probe 1	IM Q-probe 2	IM Q-probe 3		
*C. albicans*	61.8	65.3	59.2	0	*C. albicans*
*C. glabrata*	—	55.0	58.0	0.14	*C. glabrata*
*C. parapsilosis*	54.0	65.7	56.0	0.22	*C. parapsilosis*
*C. tropicalis*	54.0	65.4	59.7	0.41	*C. tropicalis*
*C. krusei*	53.2	59.3	59.3	0.22	*C. krusei*
*C. lusitaniae*	—	59.0	62.3	0.14	*C. lusitaniae*
*C. dubliniensis*	57.8	65.2	59.3	0.33	*C. dubliniensis*
*C. guilliermondii*	50.7	65.5	62.5	0.22	*C. guilliermondii*

*These *Candida* species were identified using the sequencing method.

Finally, using 16 whole blood samples (2 mL) collected from candidemia patients, we assessed the accuracy of the IM Q-probe method compared that of the culture/sequencing method. The individual IM Q-probe results compared with the culture and sequencing results are shown in Table 5. Of the total of 16 patient samples, 100% (16/16) matched the culture and sequencing results with D values of ≤0.41. The breakdown of the identification results was, 11 cases of *C. albicans*, 4 cases of *C. parapsilosis*, and 1 case of *C. tropicalis*.

**Table 5:** Individual results of identification starting from whole blood samples of candidemia patients.

Patient	Identification results				Match	
	Conventional culture method	Sequencing method	IM Q-probe method	D Value	Cult.	Seq.
HC	Not detected	Not detected	Not detected	—	—	—
1	*C. albicans*	*C. albicans*	*C. albicans*	0.22	✓	✓
2	*C. albicans*	*C. albicans*	*C. albicans*	0.37	✓	✓
3	*C. albicans*	*C. albicans*	*C. albicans*	0.22	✓	✓
4	*C. albicans*	*C. albicans*	*C. albicans*	0.22	✓	✓
5	*C. albicans*	*C. albicans*	*C. albicans*	0.36	✓	✓
6	*C. albicans*	*C. albicans*	*C. albicans*	0.22	✓	✓
7	*C. albicans*	*C. albicans*	*C. albicans*	0.14	✓	✓
8	*C. albicans*	*C. albicans*	*C. albicans*	0.22	✓	✓
9	*C. albicans*	*C. albicans*	*C. albicans*	0.24	✓	✓
10	*C. albicans*	*C. albicans*	*C. albicans*	0.14	✓	✓
11	*C. albicans*	*C. albicans*	*C. albicans*	0.24	✓	✓
12	*C. parapsilosis*	*C. parapsilosis*	*C. parapsilosis*	0.36	✓	✓
13	*C. parapsilosis*	*C. parapsilosis*	*C. parapsilosis*	0.37	✓	✓
14	*C. parapsilosis*	*C. parapsilosis*	*C. parapsilosis*	0.41	✓	✓
15	*C. parapsilosis*	*C. parapsilosis*	*C. parapsilosis*	0.14	✓	✓
16	*C. tropicalis*	*C. tropicalis*	*C. tropicalis*	0.22	✓	✓

HC, healthy control; Cult., culture results; Seq., sequencing results.

✓IM Q-probe identification result matched the culture/sequencing result.

## Discussion

In relation to fungal infection, rapid identification methods of *Candida* species using a high-resolution melting-curve (HRM) analysis have been previously reported^12–14^. None of those reports used whole blood samples collected from candidemia patients to assess the accuracy of their rapid identification methods. HRM analyses primarily rely on differences in the melting curve shapes. The IM Q-probe method does not use melting curve shapes, instead relying only on Tm values. For this reason, although melting curve shapes are affected by various DNA concentrations^15^, Tm values are not. In fact, fungal concentrations in candidemia vary among individual patients.

Mylonakis *et al*. reported the utility of a T2 magnetic resonance (T2MR) assay for the rapid diagnosis of candidemia in whole blood^16,17^. Using a T2MR assay, *Candida* species can be detected directly from a clinical sample with high sensitivity. However, the T2MR assay detects only five kinds of *Candida* species (*C. albicans*, *C. glabrata*, *C. parapsilosis*, *C. tropicalis*, and *C. krusei*). Furthermore, to identify the *Candida* species, gene sequencing analyses are additionally needed. In general, gene sequencing analyses tend to be costly and therefore have limited applications for identifying pathogens in clinical practice.

Employing only one *Candida* universal primer and three probes, the IM Q-probe method enabled the rapid detection and identification of the eight *Candida* species within three and a half hours of whole blood collection. These findings suggest that this method is rapid and easy for identifying pathogenic *Candida* species in cases of candidemia. If different-colored fluorescent IM Q-probes are employed, multiplexing in a single tube might be possible, which equates to even easier performance.

In order to identify various *Candida* species, we designed quenching probes with some probe-target mismatches. Based on the nearest neighbor thermodynamic theory^18,19^, the variations in melting temperature of the IM Q-probes depend on the number and position of probe-target mismatches on IM Q-probe hybridization. For this reason, the Tm values of the IM Q-probes provided adequate diversity for the IM Q-probe method to identify the eight *Candida* species at the species level. Chakravorty *et al*. reported the rapid identification of bacterial isolates using a sloppy molecular beacon (SMB) melting temperature signature technique^20^. SMB probes possess relatively long probe sequences, enabling them to form hybrids with PCR amplicons despite the presence of mismatched base pairs. However, we designed the imperfect-match “Q probes” so as not to form secondary structures, since Q probes tend to self-quench by forming secondary structures. To obtain stable identification results, we selected IM Q-probe positions whose target base sequence was the same among different strains of the same *Candida* species. If the IM Q-probe method identifies a mutant strain (D value > 0.41) with a mutation in the IM Q-probe target regions, we will register it in the database. Consequently, the IM Q-probe method would be able to identify mutant strain as well. In addition, to obtain stable identification results, the pH and salt concentration of the PCR buffer should be consistent. Therefore, preparing a reagent kit is advised.

We first set the interpretative criteria to assess the suitability of the IM Q-probe identification. Considering the maximum measurement error of the IM Q-probes (±0.25 °C) using Rotor-Gene Q instrument whose tube-to-tube variation was within ±0.1 °C, the identification results with a D value ≤ 0.41 were considered the *Candida* isolate. This interpretative criterion with the D value can be revised according to the maximum measurement error, and the tube-to-tube variation caused by each real-time PCR instrument is the main component of the maximum measurement error. The tube-to-tube variations in almost all commercially available real-time PCR instruments are within ±0.3 to ±0.5 °C. If the maximum measurement error is within ±0.8 °C, then the range in the theoretical D value should be within 1.31. Considering this range of D value and the high specificity of the IM Q-probe method (Table S4 shows the D value between the most similar *Candida* species in the database is 2.98), almost all commercially available real-time PCR instruments can feasibly be used for the IM Q-probe method to correctly identify any of eight *Candida* species in a clinical sample.

Concerning the specificity of the IM Q-probe method, the method detects not only the eight *Candida* species (*C. albicans*, *C. glabrata*, *C. parapsilosis*, *C. tropicalis*, *C. krusei*, *C. lusitaniae*, *C. dubliniensis*, *C. guilliermondii*) but also other kinds of *Candida* species (*C. fermentati*, *C. inconspicua*, *C. kefyr*, *C. nivariensis*, *C. norvegensis*, *C. orthopsilosis*, *C. pelliculosa*, *C. sake*, *C. zeylanoides*, etc.) since the *Candida* universal primer can detect all species of *Candida*. In this regard, the problem is that *C. tropicalis* and *C. orthopsilosis* cannot be distinguished using the IM Q-probe method (Table S6). Likewise, *C. guilliermondii*, *C. fermentati* and *C. zeylanoides* cannot be distinguished as well. However, the frequency of other kinds of *Candida* species causing candidemia is only 0.8% (Table S6), so this does not appear to present an issue in identifying *Candida* species in clinical practice. Of note, regarding specificity, the IM Q-probe method can also identify genus *Aspergillus* and genus *Cryptococcus* at the genus level (Table S7). It is easy to distinguish genus *Candida* from genus *Aspergillus* and genus *Cryptococcus* due to the high specificity of the IM Q-probe identification. However, the purpose of the IM Q-probe method is to identify any of eight *Candida* species in a clinical sample. In order to identify various kinds of fungi at the species level, additional developments will be required.

In conclusion, the IM Q-probe method enabled the rapid identification of eight *Candida* species in a clinical sample within three and a half hours of whole blood collection. Furthermore, using the D value, it was possible to quickly identify samples suitable for IM Q-probe identification. The IM Q-probe method is expected to contribute not only to the life expectancy of candidemia patients but also to antifungal stewardship.

## Methods

### *Candida* isolates

*Candida* isolates were obtained from patient’s blood, sputum and urine culture isolates at Toyama University Hospital, then were sequenced and identified at the species level.

### Clinical samples

A total of 16 whole blood samples were collected from patients with candidemia at Toyama University Hospital. All procedures were performed under a protocol approved by the Ethics Committee of the University of Toyama, and written informed consent was obtained from all patients. The methods were carried out according to the approved guidelines.

### Isolation of *candida* genomic DNA from colonies

The *Candida* colonies were selected with a sterile inoculating loop and suspended in 1 mL of molecular-grade distilled water (water deionized and sterilized for molecular biology; NACALAI TESQUE, INC., Kyoto, Japan). The sample was subsequently centrifuged at 20,000 × g for 10 minutes, and 900 μL of the supernatant was carefully removed so as not to lose the pellet. DNA was isolated from the resulting pellets using a DNA extraction kit (QIAamp UCP Pathogen Mini Kit; Qiagen, Hilden, Germany) and glass beads (Pathogen Lysis Tubes S; Qiagen) in accordance with the supplier’s instructions. Finally, *Candida* DNA was eluted with 100 μL of elution buffer.

### Isolation of *candida* genomic DNA from whole blood

A total of 2 mL of venous blood was collected in EDTA-2K tube (NIPRO, Osaka, Japan). The blood sample was then centrifuged at 100 × g for 5 minutes to spin down the blood cells, and the resulting supernatant fraction (1 mL) was used. The supernatant was centrifuged again at 20,000 × g for 10 minutes, and 900 μL of the supernatant fraction was carefully removed so as not to disturb the pellet. Next, 1 mL of molecular-grade distilled water (water deionized and sterilized for molecular biology; NACALAI TESQUE, INC.) was added to the pellet, and the mixture was gently turned upside down several times, and subsequently centrifuged again at 20,000 × g for 5 minutes. Finally, 1 mL of the supernatant fraction was again carefully removed unless you resuspend the pellet. DNA was isolated from the pellet employing a DNA extraction kit (QIAamp UCP Pathogen Mini Kit; Qiagen) and glass beads (Pathogen Lysis Tubes S; Qiagen) in accordance with the supplier’s instructions. Finally, *Candida* DNA was eluted with 100 μL of elution buffer.

### PCR assays

In each of the following processes, the QIAgility system (Qiagen) provided an automated PCR setup. The Rotor-Gene Q (Qiagen) was used for the amplification, real-time detection of the target DNA and Tm value analysis of the Imperfect Match Quenching probes (IM Q-probes). All PCR assays were performed as single-tube assays (no multiplex PCR). We used 1.5 mL PCR-clean Eppendorf tubes that were RNase- and DNase-free (Eppendorf, Hamburg, Germany) and 0.2 mL PCR tubes (Qiagen) for PCR. Fungal (*Candida*) universal primer targeting the 18 S ribosomal RNA gene (18 S rDNA), which has been described previously^21^, were synthesized by Life Technologies Japan, Ltd. (Tokyo, Japan). The IM Q-probes were designed using a multiple alignment software program (Clustal X) and were synthesized by NIPPON STEEL and SUMIKIN Eco-Tech Corporation (Ibaragi, Japan). The IM Q-probes were as follows: IM Q-probe 1 (5′-CTTTCCTTCTGGGTAGCCATTT-3′, probe size: 22 bp), IM Q-probe 2 (5′-TGGAATAATAGAATAGGACGTTATGGTTC-3′, probe size: 29 bp), IM Q-probe 3 (5′-GCATCAGTAATCAGTTGTCAGAGGAGAAATTC-3′, probe size: 32 bp).

During the PCR procedure, the PCR reaction mixture (50 μL) contained 7 μL of DNA template in KOD FX Neo reagents (1 × PCR Buffer with 2.0 mM of Mg^2+^, 0.4 mM of dNTPs and 0.02 U of KOD FX Neo → 1.0 U of KOD FX Neo U of KOD FX Neo; TOYOBO LIFE SCIENCE, Osaka, Japan), 0.2 μM of *Candida* universal forward primer, 0.6 μM of *Candida* universal reverse primer and 0.02 μM of each IM Q-probe.

Each sample was incubated for 5 minutes at 95 °C, then was denatured for 10 seconds at 98 °C, annealed for 30 seconds at 57 °C, extended for 20 seconds at 72 °C and subjected to fluorescence acquisition for 2 seconds at 82 °C for 50 cycles.

### The Tm value analysis

For the Tm value analysis, the resulting amplicons with each IM Q-probe were heated at 95 °C for 10 seconds and then cooled at 40 °C for 90 seconds. A post-PCR Tm value analysis of the 3 probes was performed from 40 °C to 95 °C, increasing at 1 °C/step. The data profile was subsequently analyzed using the Rotor-Gene Q software program, and the Tm values were identified.

### Analytical sensitivity tests

Prior to performing the sensitivity tests, *C. albicans* was cultivated in Sabouraud dextrose broth at 37 °C for 48 h, and *C. albicans* suspensions (1 mL) in phosphate-buffered saline (PBS) were prepared. A total of 50 µL of the diluted suspensions were inoculated on Sabouraud dextrose agar. After incubation at 37 °C for 24 h, the number of colony-forming units (CFU) was determined by counting the colonies grown on the agar plates in triplicate^12^.

The limits of identification and detection were determined by serially diluting cultures with known counts (CFU) of *C. albicans* in PBS and subjecting the samples to IM Q-probe identification using the IM Q-probes. The limit of identification was determined to be the final dilution of the template in which the IM Q-probe identification result was correct (Table S5) with a D value of ≤0.41.

### A nucleotide sequence-based analysis of fungal genomic DNA

PCR amplicons amplified the D1-D2 region and the internal transcribed spacer (ITS) regions using fungal universal primers were purified (QIAquick PCR Purification Kit; QIAGEN) and then sequenced (3500 Genetic Analyzer; Thermo Fisher Scientific, Waltham, Massachusetts, USA)^22^. An online homology search was performed for strain identification using the BLAST nucleotide database tool of the DNA Data Bank of Japan (http://www.ddbj.nig.ac.jp/index-j.html).

### Culture-based biochemical identification of *Candida* species

The whole blood samples (one aerobic blood culture bottle and one anaerobic blood culture bottle) were collected simultaneously with the blood sample for the IM Q-probe analysis from the same puncture site. The whole blood samples were then analyzed according to the standard methods used by the Clinical Laboratory Center (certified ISO15189) at Toyama University Hospital. The blood culture procedures were performed using the BacT/ALERT 3D system (bioMérieux, Inc., Marcy-l'Étoile, France). Identification of fungi was presumptively performed by CHROMager^TM^ Candida (Becton Dickinson and Company, Franklin Lakes, New Jersey, USA) and definitely performed by matrix-assisted laser desorption ionization-time of flight (MALDI-TOF) mass spectrometry (Bruker, Billerica, Massachusetts, USA).

## Supplementary information

Supplementary Information.
