# Supplementary material for: Rapid Identification of Candida Species in Candidemia Directly from Blood Samples Using Imperfect Match Probes
Source: Sci Rep. 2020 Apr 2;10:5828. doi: 10.1038/s41598-020-62276-5 (PMC7118160; doi:10.1038/s41598-020-62276-5)
Supplement: Supplementary file 1 — Supplementary Information. [file 41598_2020_62276_MOESM1_ESM.docx]

***Supplementary Information***

**Rapid Identification of *Candida* Species in Candidemia Directly from Blood Samples Using Imperfect Match Probes**

Yoshitsugu Higashi^1^†, Hideki Niimi^2*^†, Ippei Sakamaki^1^, Yoshihiro Yamamoto^1^

and Isao Kitajima^2^

1. Department of Clinical Infectious Diseases, Graduate School of Medicine and Pharmaceutical Sciences for Research, University of Toyama, Toyama 930-0194, Japan
2. Department of Clinical Laboratory and Molecular Pathology, Graduate School of Medicine and Pharmaceutical Sciences for Research, University of Toyama, Toyama 930-0194, Japan

*correspondence to:

Hideki Niimi, M.D. Ph.D. E-mail address: hiniimi@med.u-toyama.ac.jp

Department of Clinical Laboratory and Molecular Pathology, Graduate School of Medicine and Pharmaceutical Sciences for Research, University of Toyama,

2630 Sugitani, Toyama 930-0194, JAPAN

Phone: (+81)-76-434-7759, Fax: (+81)-76-434-7759

†Both authors contributed equally to this work

**Table S1. Sequence homology between the IM Q-probes and the target regions of the eight *Candida* species reported in the DNA Data Bank of Japan**

| ***Candida* species** | accession No. | **IM Q-probe 1** | | |
| --- | --- | --- | --- | --- |
|  |  | sequence (5′→3′) | probe position | Mismatch |
|  |  | CTTTCCTTCTGGGTAGCCATTT |  |  |
| *C. albicans* | AF114470 | CTTTCCTTCTGGGTAGCCATTT | 585-607 | 0 |
|  | AY497744 | CTTTCCTTCTGGGTAGCCATTT | 548-570 | 0 |
|  | JN940588 | CTTTCCTTCTGGGTAGCCATTT | 617-639 | 0 |
|  | JN941105 | CTTTCCTTCTGGGTAGCCATTT | 690-712 | 0 |
| *C. glabrata* | AB094140 | CTTTCCTTCTGGCTAACCCCAA | 686-708 | 6 |
|  | AY046237 | CTTTCCTTCTGGCTAACCCCAA | 670-692 | 6 |
|  | AY083231 | CTTTCCTTCTGGCTAACCCCAA | 577-599 | 6 |
|  | KT229542 | CTTTCCTTCTGGCTAACCCCAA | 664-686 | 6 |
| *C. parapsilosis* | AB030915 | CTTTCCTTCTGGCTAGCCTTTT | 647-669 | 2 |
|  | AY055857 | CTTTCCTTCTGGCTAGCCTTTT | 690-712 | 2 |
|  | JQ008832 | CTTTCCTTCTGGCTAGCCTTTT | 619-641 | 2 |
|  | KF255835 | CTTTCCTTCTGGCTAGCCTTTT | 448-470 | 2 |
| *C. tropicalis* | EF412966 | CTTTCCTTCTGGCTAGCCTTTT | 647-669 | 2 |
|  | EU034726 | CTTTCCTTCTGGCTAGCCTTTT | 690-712 | 2 |
|  | JQ008834 | CTTTCCTTCTGGCTAGCCTTTT | 617-639 | 2 |
|  | KT449837 | CTTTCCTTCTGGCTAGCCTTTT | 623-645 | 2 |
| *C. krusei* | AB053239 | CTTTCCTTCTGGCTAGCCCTCG | 659-681 | 4 |
|  | EF550360 | CTTTCCTTCTGGCTAGCCCTCG | 625-647 | 4 |
|  | JF274497 | CTTTCCTTCTGGCTAGCCCTCG | 638-660 | 4 |
|  | KU147485 | CTTTCCTTCTGGCTAGCCCTCG | 420-442 | 4 |
| *C. lusitaniae* | AY497762 | CTTTCCTCCTCCTCTTAGCAAT | 527-549 | 12 |
|  | FJ176816 | CTTTCCTCCTCCTCTTAGCAAT | 650-672 | 12 |
|  | JN941111 | CTTTCCTCCTCCTCTTAGCAAT | 674-696 | 12 |
|  | JQ698900 | CTTTCCTCCTCCTCTTAGCAAT | 639-661 | 12 |
| *C. dubliniensis* | AY497766 | CTTTCCTTCTGGCTAGCCATTT | 548-570 | 1 |
|  | AY669334 | CTTTCCTTCTGGCTAGCCATTT | 119-141 | 1 |
|  | MF045510 | CTTTCCTTCTGGCTAGCCATTT | 148-170 | 1 |
|  | FM992695 | CTTTCCTTCTGGCTAGCCATTT | 1864058-1864080 | 1 |
| *C. guilliermondii* | AB054282 | CTTTCCTTCTGGCTAACCATTC | 666-688 | 3 |
|  | AY227020 | CTTTCCTTCTGGCTAACCATTC | 624-646 | 3 |
|  | HG798649 | CTTTCCTTCTGGCTAACCATTC | 668-690 | 3 |
|  | KJ126853 | CTTTCCTTCTGGCTAACCATTC | 691-713 | 3 |

| ***Candida* species** | accession No. | **IM Q-probe 2** | | |
| --- | --- | --- | --- | --- |
|  |  | sequence (5′→3′) | probe position | Mismatch |
|  |  | TGGAATAATAGAATAGGACGTTATGGTTC |  |  |
| *C. albicans* | AF114470 | TGGAATAATAGAATAGGACGTTATGGTTC | 679-708 | 0 |
|  | AY497744 | TGGAATAATAGAATAGGACGTTATGGTTC | 642-671 | 0 |
|  | JN940588 | TGGAATAATAGAATAGGACGTTATGGTTC | 711-740 | 0 |
|  | JN941105 | TGGAATAATAGAATAGGACGTTATGGTTC | 784-813 | 0 |
| *C. glabrata* | AB094140 | TGGAATAATGGAATAGGACGTT-TGGTTC | 795-823 | 2 |
|  | AY046237 | TGGAATAATGGAATAGGACGTT-TGGTTC | 779-808 | 2 |
|  | AY083231 | TGGAATAATGGAATAGGACGTT-TGGTTC | 686-715 | 2 |
|  | KT229542 | TGGAATAATGGAATAGGACGTT-TGGTTC | 774-803 | 2 |
| *C. parapsilosis* | AB030915 | TGGAATAATAGAATAGGACGTTATGGTTC | 741-770 | 0 |
|  | AY055857 | TGGAATAATAGAATAGGACGTTATGGTTC | 790-819 | 0 |
|  | JQ008832 | TGGAATAATAGAATAGGACGTTATGGTTC | 719-748 | 0 |
|  | KF255835 | TGGAATAATAGAATAGGACGTTATGGTTC | 544-573 | 0 |
| *C. tropicalis* | EF412966 | TGGAATAATAGAATAGGACGTTATGGTTC | 738-767 | 0 |
|  | EU034726 | TGGAATAATAGAATAGGACGTTATGGTTC | 782-811 | 0 |
|  | JQ008834 | TGGAATAATAGAATAGGACGTTATGGTTC | 707-736 | 0 |
|  | KT449837 | TGGAATAATAGAATAGGACGTTATGGTTC | 715-744 | 0 |
| *C. krusei* | AB053239 | TGGAATAATAGAATAGGACGC-ATGGTTC | 751-779 | 2 |
|  | EF550360 | TGGAATAATAGAATAGGACGC-ATGGTTC | 717-745 | 2 |
|  | JF274497 | TGGAATAATAGAATAGGACGC-ATGGTTC | 730-758 | 2 |
|  | KU147485 | TGGAATAATAGAATAGGACGC-ATGGTTC | 512-540 | 2 |
| *C. lusitaniae* | AY497762 | TGGAATAATAGAATAGGACGC-ATGGTTC | 618-646 | 2 |
|  | FJ176816 | TGGAATAATAGAATAGGACGC-ATGGTTC | 741-769 | 2 |
|  | JN941111 | TGGAATAATAGAATAGGACGC-ATGGTTC | 766-794 | 2 |
|  | JQ698900 | TGGAATAATAGAATAGGACGC-ATGGTTC | 730-758 | 2 |
| *C. dubliniensis* | AY497766 | TGGAATAATAGAATAGGACGTTATGGTTC | 642-671 | 0 |
|  | AY669334 | TGGAATAATAGAATAGGACGTTATGGTTC | 213-242 | 0 |
|  | MF045510 | TGGAATAATAGAATAGGACGTTATGGTTC | 242-271 | 0 |
|  | FM992695 | TGGAATAATAGAATAGGACGTTATGGTTC | 1864151-1864180 | 0 |
| *C. guilliermondii* | AB054282 | TGGAATAATAGAATAGGACGTTATGGTTC | 773-802 | 0 |
|  | AY227020 | TGGAATAATAGAATAGGACGTTATGGTTC | 731-760 | 0 |
|  | HG798649 | TGGAATAATAGAATAGGACGTTATGGTTC | 775-804 | 0 |
|  | KJ126853 | TGGAATAATAGAATAGGACGTTATGGTTC | 798-827 | 0 |

| ***Candida* species** | accession  No. | **IM Q-probe 3** | | |  |
| --- | --- | --- | --- | --- | --- |
|  |  | sequence (5′→3′) | probe position | Mismatch | |
|  |  | GCATCAGTAATCAGTTGTCAGAGGAGAAATTC |  |  |  |
| *C. albicans* | AF114470 | GTATCAGTATTCAGTTGTCAGAGGTGAAATTC | 758-790 | 3 | |
|  | AY497744 | GTATCAGTATTCAGTTGTCAGAGGTGAAATTC | 721-753 | 3 | |
|  | JN940588 | GTATCAGTATTCAGTTGTCAGAGGTGAAATTC | 791-823 | 3 | |
|  | JN941105 | GTATCAGTATTCAGTTGTCAGAGGTGAAATTC | 864-896 | 3 | |
| *C. glabrata* | AB094140 | GCATCAGTATTCAATTGTCAGAGGTGAAATTC | 874-906 | 3 | |
|  | AY046237 | GCATCAGTATTCAATTGTCAGAGGTGAAATTC | 858-890 | 3 | |
|  | AY083231 | GCATCAGTATTCAATTGTCAGAGGTGAAATTC | 765-797 | 3 | |
|  | KT229542 | GCATCAGTATTCAATTGTCAGAGGTGAAATTC | 852-884 | 3 | |
| *C. parapsilosis* | AB030915 | GTATCAGTATTCAGTAGTCAGAGGTGAAATTC | 820-852 | 4 | |
|  | AY055857 | GTATCAGTATTCAGTAGTCAGAGGTGAAATTC | 863-895 | 4 | |
|  | JQ008832 | GTATCAGTATTCAGTAGTCAGAGGTGAAATTC | 792-824 | 4 | |
|  | KF255835 | GTATCAGTATTCAGTAGTCAGAGGTGAAATTC | 621-653 | 4 | |
| *C. tropicalis* | EF412966 | GTATCAGTATTCAGTTGTCAGAGGTGAAATTC | 819-851 | 3 | |
|  | EU034726 | GTATCAGTATTCAGTTGTCAGAGGTGAAATTC | 862-894 | 3 | |
|  | JQ008834 | GTATCAGTATTCAGTTGTCAGAGGTGAAATTC | 789-821 | 3 | |
|  | KT449837 | GTATCAGTATTCAGTTGTCAGAGGTGAAATTC | 795-827 | 3 | |
| *C. krusei* | AB053239 | GCATCAGTATTCAGTCGTCAGAGGTGAAATTC | 830-862 | 3 | |
|  | EF550360 | GCATCAGTATTCAGTCGTCAGAGGTGAAATTC | 796-828 | 3 | |
|  | JF274497 | GCATCAGTATTCAGTCGTCAGAGGTGAAATTC | 809-841 | 3 | |
|  | KU147485 | GCATCAGTATTCAGTCGTCAGAGGTGAAATTC | 591-623 | 3 | |
| *C. lusitaniae* | AY497762 | GCATCAGTATTCAGTTGTCAGAGGTGAAATTC | 696-718 | 2 | |
|  | FJ176816 | GCATCAGTATTCAGTTGTCAGAGGTGAAATTC | 819-851 | 2 | |
|  | JN941111 | GCATCAGTATTCAGTTGTCAGAGGTGAAATTC | 844-876 | 2 | |
|  | JQ698900 | GCATCAGTATTCAGTTGTCAGAGGTGAAATTC | 808-840 | 2 | |
| *C. dubliniensis* | AY497766 | GTATCAGTATTCAGTTGTCAGAGGTGAAATTC | 722-754 | 3 | |
|  | AY669334 | GTATCAGTATTCAGTTGTCAGAGGTGAAATTC | 293-325 | 3 | |
|  | MF045510 | GTATCAGTATTCAGTTGTCAGAGGTGAAATTC | 322-354 | 3 | |
|  | FM992695 | GTATCAGTATTCAGTTGTCAGAGGTGAAATTC | 1864232-1864264 | 3 | |
| *C. guilliermondii* | AB054282 | GCATCAGTATTCAGTTGTCAGAGGTGAAATTC | 853-885 | 2 | |
|  | AY227020 | GCATCAGTATTCAGTTGTCAGAGGTGAAATTC | 811-843 | 2 | |
|  | HG798649 | GCATCAGTATTCAGTTGTCAGAGGTGAAATTC | 855-887 | 2 | |
|  | KJ126853 | GCATCAGTATTCAGTTGTCAGAGGTGAAATTC | 878-910 | 2 | |

The base sequence differences between the IM Q-probes and the target regions are shown in red.

**Table S2. The Tm value database of the eight *Candida* species**

| *Candida* species | Tm degree (℃) | | |
| --- | --- | --- | --- |
|  | IM Q-probe 1 | IM Q-probe 2 | IM Q-probe 3 |
| *C. albicans* | 62.0 | 65.5 | 59.4 |
| *C. glabrata* | - | 55.0 | 57.8 |
| *C. parapsilosis* | 53.5 | 65.5 | 55.6 |
| *C. tropicalis* | 53.7 | 65.6 | 59.4 |
| *C. krusei* | 53.3 | 59.2 | 59.1 |
| *C. lusitaniae* | - | 59.3 | 62.4 |
| *C. dubliniensis* | 58.2 | 65.6 | 59.3 |
| *C. guilliermondii* | 50.3 | 65.4 | 62.2 |

Using the mean value of triplicate Tm value measurements, we constructed a preliminary database of the eight *Candida* species, all of which are detected in our hospital.

**Table S3. Validation of the measurement errors among 10 samples using the same *Candida albicans* DNA template in the same trial**

| Sample | Tm degree (℃) | | |
| --- | --- | --- | --- |
|  | IM Q-Probe 1 | IM Q-Probe 2 | IM Q-Probe 3 |
| 1 | 61.8 | 65.0 | 59.3 |
| 2 | 62.0 | 65.3 | 59.5 |
| 3 | 62.0 | 65.3 | 59.5 |
| 4 | 62.0 | 65.3 | 59.5 |
| 5 | 62.2 | 65.3 | 59.5 |
| 6 | 62.2 | 65.5 | 59.5 |
| 7 | 62.2 | 65.5 | 59.5 |
| 8 | 62.2 | 65.5 | 59.5 |
| 9 | 62.2 | 65.5 | 59.5 |
| 10 | 62.2 | 65.5 | 59.7 |
| mean | 62.1 | 65.37 | 59.5 |
| SD | 0.14 | 0.16 | 0.09 |
| CV | 0.002 | 0.002 | 0.002 |
| Max | 62.2 | 65.5 | 59.7 |
| Min | 61.8 | 65.0 | 59.3 |
| Range | 62.0 ± 0.2 | 65.25 ± 0.25 | 59.5 ± 0.2 |

**Table S4. The specificity with D values (calculated using the registered Tm values) among the eight *Candida* species in the database**

| *Candida* species in the database | most similar *Candida* species | D Value |
| --- | --- | --- |
| *C. albicans* | *C. dubliniensis* | 3.11 |
| *C. glabrata* | *C. lusitaniae* | 0.21 |
| *C. parapsilosis* | *C. tropicalis* | 2.98 |
| *C. tropicalis* | *C. parapsilosis* | 2.98 |
| *C. krusei* | *C. dubliniensis* | 3.71 |
| *C. lusitaniae* | *C. glabrata* | 0.21 |
| *C. dubliniensis* | *C. albicans* | 3.11 |
| *C. guilliermondii* | *C. tropicalis* | 4.38 |

**Table S5. Validation of the limit of identification in triplicate trials using the same**

***C. albicans* DNA template**

| CFU/  PCR Tube | Trial | Tm degree (℃) | | | D Value | Identification results |
| --- | --- | --- | --- | --- | --- | --- |
|  |  | IM Q-probe 1 | IM Q-probe 2 | IM Q-probe 3 |  |  |
| 88 | 1st | 62.5 | 66.5 | 60.0 | 0.374 | *C. albicans* |
|  | 2nd | 62.8 | 66.5 | 60.0 | 0.283 | *C. albicans* |
|  | 3rd | 62.7 | 66.0 | 60.0 | 0.141 | *C. albicans* |
| 1.75 | 1st | 62.5 | 65.8 | 59.7 | 0.163 | *C. albicans* |
|  | 2nd | 63.0 | 66.5 | 60.0 | 0.327 | *C. albicans* |
|  | 3rd | 62.5 | 66.0 | 60.0 | 0.08 | *C. albicans* |
| 1.2 | 1st | 62.7 | 66.0 | 59.8 | 0.22 | *C. albicans* |
|  | 2nd | 63.0 | 66.0 | 60.0 | 0.37 | *C. albicans* |
|  | 3rd | 63.0 | 66.0 | 60.0 | 0.37 | *C. albicans* |
| 0.88 | 1st | 62.8 | - | - | - | Not identified |
|  | 2nd | 63.0 | - | 60.0 | - | Not identified |
|  | 3rd | - | - | 60.0 | - | Not identified |

CFU, colony forming unit

-: not detected

**Table S6. Sequence homology between the IM Q-probes and fungal target regions**

| *Candida* species |  | Freq.  (%) | **IM Q-probe 1** (5′→3′) | mismatch  (bp) |
| --- | --- | --- | --- | --- |
|  |  |  | CTTTCCTTCTGGGTAGCCATTT |  |
| *Candida albicans* | (AF114470) | 42.1 | CTTTCCTTCTGGGTAGCCATTT | 0 |
| *Candida glabrata* | (AY083231) | 26.7 | CTTTCCTTCTGGCTAACCCCAA | 6 |
| *Candida parapsilosis* | (AB030915) | 15.9 | CTTTCCTTCTGGCTAGCCTTTT | 2 |
| *Candida tropicalis**^1^ | (KT449837) | 8.7 | CTTTCCTTCTGGCTAGCCTTTT | 2 |
| *Candida krusei* | (AB053239) | 3.4 | CTTTCCTTCTGGCTAGCCCTCG | 4 |
| *Candida lusitaniae* | (KU147480) | 1.1 | CTTTCCTCCTCCTCTTAGCAAT | 12 |
| *Candida dubliniensis* | (AY497766) | 0.9 | CTTTCCTTCTGGCTAGCCATTT | 1 |
| *Candida guilliermondii**^2^ | (AY497770) | 0.4 | CTTTCCTTCTGGCTAACCATTC | 3 |
| *Candida fermentati**^2^ | (AY553853) |  | CTTTCCTTCTGGCTAACCATTC | 3 |
| *Candida inconspicua* | (AB053243) |  | CTTTCCTTCTGGCTAGCCTTCG | 4 |
| *Candida kefyr* | (AB054675) |  | CTTTCCTTCTGGCTAACCTTGT | 4 |
| *Candida nivariensis* | (AY727047) |  | CTTTCCTTCTGGCTAACCCCAA | 6 |
| *Candida norvegensis* | (AB053237) | 0.8 | CTTTCCTTCTGGCTAGCCTCCC | 5 |
| *Candida orthopsilosis**^1^ | (HQ215535) |  | CTTTCCTTCTGGCTAGCCTTTT | 2 |
| *Candida pelliculosa* | (AB054562) |  | CTTTCCTTCTGGCTAACCTGTC | 5 |
| *Candida sake* | (AB013529) |  | CTTTCCTTCTGGGTAACCTTTC | 3 |
| *Candida zeylanoides**^2^ | (AB013509) |  | CTTTCCTTCTGGCTAACCATTC | 3 |

| *Candida* species |  | Freq.  (%) | **IM Q-probe 2** (5′→3′) | mismatch  (bp) |
| --- | --- | --- | --- | --- |
|  |  |  | TGGAATAATAGAATAGGACGTTATGGTTC |  |
| *Candida albicans* | (AF114470) | 42.1 | TGGAATAATAGAATAGGACGTTATGGTTC | 0 |
| *Candida glabrata* | (AY083231) | 26.7 | TGGAATAATGGAATAGGACGTT-TGGTTC | 2 |
| *Candida parapsilosis* | (AB030915) | 15.9 | TGGAATAATAGAATAGGACGTTATGGTTC | 0 |
| *Candida tropicalis**^1^ | (KT449837) | 8.7 | TGGAATAATAGAATAGGACGTTATGGTTC | 0 |
| *Candida krusei* | (AB053239) | 3.4 | TGGAATAATAGAATAGGACGC-ATGGTTC | 2 |
| *Candida lusitaniae* | (KU147480) | 1.1 | TGGAATAATAGAATAGGACGC-ATGGTTC | 2 |
| *Candida dubliniensis* | (AY497766) | 0.9 | TGGAATAATAGAATAGGACGTTATGGTTC | 0 |
| *Candida guilliermondii**^2^ | (AY497770) | 0.4 | TGGAATAATAGAATAGGACGTTATGGTTC | 0 |
| *Candida fermentati**^2^ | (AY553853) |  | TGGAATAATAGAATAGGACGTTATGGTTC | 0 |
| *Candida inconspicua* | (AB053243) |  | TGGAATAATAGAATAGGACGTTATGGTTC | 0 |
| *Candida kefyr* | (AB054675) |  | TGGAATAATGGGATAGGACGT-TTGGTTC | 4 |
| *Candida nivariensis* | (AY727047) |  | TGGAATAATGGAATAGGACGT-TTGGTTC | 3 |
| *Candida norvegensis* | (AB053237) | 0.8 | TGGAATAATAGAATAGGACGTTATGGTTC | 0 |
| *Candida orthopsilosis**^1^ | (HQ215535) |  | TGGAATAATAGAATAGGACGTTATGGTTC | 0 |
| *Candida pelliculosa* | (AB054562) |  | TGGAATAATGGAATAGGACGT-TTGGTTC | 3 |
| *Candida sake* | (AB013529) |  | TGGAATAATAGAATAGGACGTTATGGTTC | 0 |
| *Candida zeylanoides**^2^ | (AB013509) |  | TGGAATAATAGAATAGGACGTTATGGTTC | 0 |

| *Candida* species |  | Freq.  (%) | **IM Q-probe 3** (5′→3′) | mismatch  (bp) |
| --- | --- | --- | --- | --- |
|  |  |  | GCATCAGTAATCAGTTGTCAGAGGAGAAATTC |  |
| *Candida albicans* | (AF114470) | 42.1 | GTATCAGTATTCAGTTGTCAGAGGTGAAATTC | 3 |
| *Candida glabrata* | (AY083231) | 26.7 | GCATCAGTATTCAATTGTCAGAGGTGAAATTC | 3 |
| *Candida parapsilosis* | (AB030915) | 15.9 | GTATCAGTATTCAGTAGTCAGAGGTGAAATTC | 4 |
| *Candida tropicalis**^1^ | (KT449837) | 8.7 | GTATCAGTATTCAGTTGTCAGAGGTGAAATTC | 3 |
| *Candida krusei* | (AB053239) | 3.4 | GCATCAGTATTCAGTCGTCAGAGGTGAAATTC | 3 |
| *Candida lusitaniae* | (KU147480) | 1.1 | GCATCAGTATTCAGTTGTCAGAGGTGAAATTC | 2 |
| *Candida dubliniensis* | (AY497766) | 0.9 | GTATCAGTATTCAGTTGTCAGAGGTGAAATTC | 3 |
| *Candida* *guilliermondii**^2^ | (AY497770) | 0.4 | GCATCAGTATTCAGTTGTCAGAGGTGAAATTC | 2 |
| *Candida* *fermentati**^2^ | (AY553853) |  | GCATCAGTATTCAGTTGTCAGAGGTGAAATTC | 2 |
| *Candida inconspicua* | (AB053243) |  | GCATCAGTATTCAGTCGTCAGAGGTGAAATTC | 3 |
| *Candida kefyr* | (AB054675) |  | GCATCAGTATTCAATTGTCAGAGGTGAAATTC | 3 |
| *Candida nivariensis* | (AY727047) |  | GCATCAGTATTCAATTGTCAGAGGTGAAATTC | 3 |
| *Candida norvegensis* | (AB053237) | 0.8 | GCATCAGTATTCAGTCGTCAGAGGTGAAATTC | 3 |
| *Candida orthopsilosis**^1^ | (HQ215535) |  | GTATCAGTATTCAGTTGTCAGAGGTGAAATTC | 3 |
| *Candida pelliculosa* | (AB054562) |  | GCATCAGTATTCAATTGTCAGAGGTGAAATTC | 3 |
| *Candida sake* | (AB013529) |  | GTATCAGTATTCAGTTGTCAGAGGTGAAATTC | 3 |
| *Candida* *zeylanoides**^2^ | (AB013509) |  | GCATCAGTATTCAGTTGTCAGAGGTGAAATTC | 2 |

The base sequence differences between the IM Q-probes and the target regions are shown in red.

*^1^*C. tropicalis* and *C. orthopsilosis* cannot be distinguished using the IM Q-probe method.

*^2^*C. guilliermondii, C. fermentati* and *C. zeylanoides* cannot be distinguished using the IM Q-probe method.

Freq. = The frequency of *Candida* species causing candidemia^10^

**Table S7. A Tm value analysis of genus *Aspergillus* and genus *Cryptococcus* using the IM Q-probe method**

| Fungi | Tm degree (℃) | | | most similar *Candida* species | D Value |
| --- | --- | --- | --- | --- | --- |
|  | IM Q-probe 1 | IM Q-probe 2 | IM Q-probe 3 |  |  |
| *Aspergillus fumigatus* | - | 56.7 | 55 | *Candida glabrata* | 3.18 |
| *Cryptococcus neoformans* | - | 56.5 | 45.2 | *Candida glabrata* | 9.97 |

-: not detected

**Figure S1. Tm value variations among eight *Candida* species with each IM Q-probe**

**
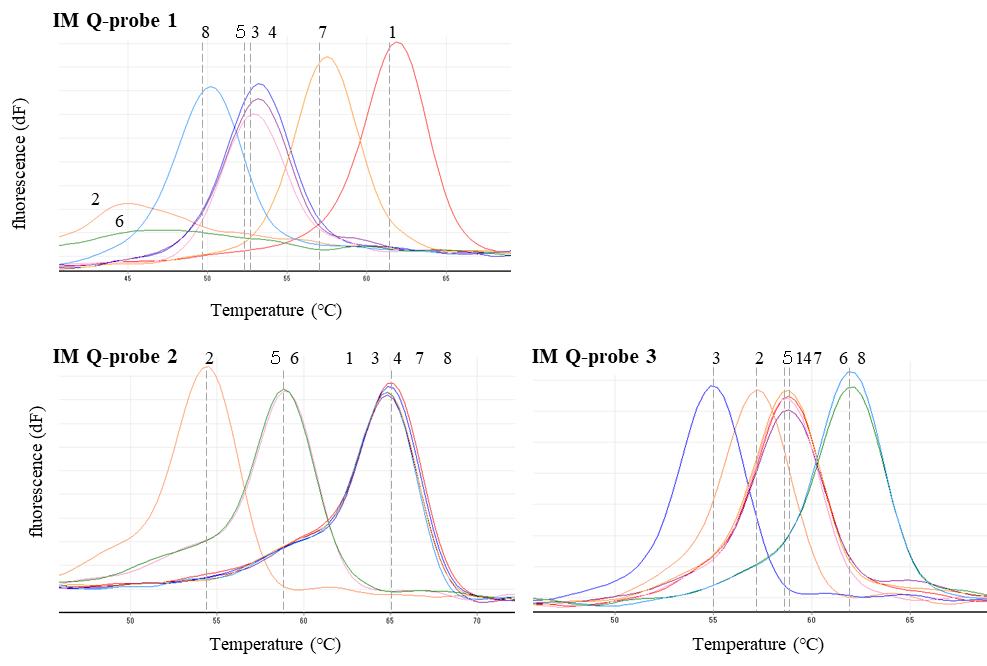
**

The Tm value variations depend on the number and position of the probe-target mismatches.

1) *C. albicans*, 2) *C. glabrata*, 3) *C. parapsilosis*, 4) *C. tropicalis*, 5) *C. krusei*, 6) *C. lusitaniae*,

7) *C. dubliniensis*, 8) *C. guilliermondii*
